# Five‐Year Changes in Nurses’ Compassion Competence and Associated Factors: A Longitudinal Study Using Explainable Machine Learning

**DOI:** 10.1155/jonm/3937997

**Published:** 2026-10-25

**Authors:** Mijin Park, Sujin Gan, EunKyo Lee, Seonghye Jo, Youngjin Lee

**Affiliations:** ^1^ Department of Nursing Kookje University Pyeongtaek South Korea; ^2^ Department of Biomedical Sciences Ajou University Graduate School of Medicine Suwon Gyeonggi–do South Korea ajou.ac.kr; ^3^ Office of Planning Hanyang IDEA Center (IR) Hanyang University Seoul South Korea hanyang.ac.kr; ^4^ College of Nursing Graduate School of Ajou University Suwon South Korea ajou.ac.kr; ^5^ College of Nursing Research Institute of Nursing Science Ajou University Suwon South Korea ajou.ac.kr

**Keywords:** compassion, longitudinal studies, machine learning, nurses, presenteeism, sleep, work environment

## Abstract

**Aims:**

To examine 5‐year changes in nurses’ compassion competence and identify personal and job‐related factors associated with these changes within the Job Demands–Resources framework.

**Background:**

Compassion competence is a dynamic professional competency shaped by individual capacities and work environments, but its long‐term change has rarely been examined.

**Methods:**

In this secondary analysis of a national Korean cohort, 332 hospital nurses who responded in 2018 (T1) and 2022 (T5) with complete data were analyzed using paired *t*‐tests, simultaneous multiple linear regression, and linear mixed‐effects growth models. Random Forest, eXtreme Gradient Boosting (XGBoost), and Light Gradient Boosting Machine (LightGBM) models were evaluated using 100 repetitions of 5‐fold cross‐validation, and feature importance was quantified using mean absolute SHapley Additive exPlanations (SHAP) values.

**Results:**

Compassion competence increased from T1 to T5 (mean change = 0.09, *p* < 0.001; 0.023 points per year in the growth model). The regression model explained 10.3% of the variance, and only baseline age was significantly (negatively) associated with change. The ensemble models showed higher in‐sample fit (*R* ^2^ = 0.33–0.41) but negative mean cross‐validated *R* ^2^ values. Changes in nurse participation in hospital affairs (positive direction) and in staffing and resource adequacy (negative direction) were the most important features in all ensemble models, with rankings and directions that were stable across folds, although their regression coefficients were not statistically significant.

**Conclusions:**

Nurses’ compassion competence increased modestly over 5 years, supporting its conceptualization as a dynamic professional competency. Nursing work environment features were the most prominent, but exploratory, model‐based contributors.

**Implications for Nursing Management:**

Nurse managers may consider nurses’ participation in hospital affairs and staffing when supporting compassionate practice; however, these factors should be evaluated in intervention studies before being adopted as management targets.

## 1. Introduction

Compassion competence is defined as nurses’ professional capacity to understand patients’ suffering and respond appropriately through therapeutic, action‐oriented care, extending beyond empathy or sympathy [1]. It encompasses cognitive understanding, emotional attunement, and behavioral responsiveness and is regarded as a foundation of therapeutic relationships and quality of care [2]. In healthcare systems characterized by increasing workloads and emotional demands, compassion requires sustained emotional labor and regulation of affective responses [3, 4] and therefore needs to be cultivated and maintained at both the individual and organizational levels.

Compassion competence is not a static trait but a dynamic, context‐sensitive competency shaped by interactions between individual capacities and work environments [1, 4]. This study adopts the Job Demands–Resources (JD‐R) model as its theoretical framework [5]. The JD‐R model posits that job resources promote motivation and engagement, whereas job demands contribute to strain and energy depletion. Within this framework, compassion competence can be conceptualized as an evolving competency that may be strengthened or eroded over time depending on the balance between available resources and demands.

From this perspective, the nursing work environment represents a prototypical job resource. Adequate staffing, participatory decision‐making, and managerial support may help preserve nurses’ psychological and emotional resources while fostering professional growth and compassionate practice, whereas inadequate environments may accelerate emotional exhaustion [6, 7]. Because such influences may accumulate over years of practice, longitudinal research is needed to clarify how changes in the work environment are associated with changes in compassion competence.

Individual health‐related conditions may also be relevant. Sleep quality provides a biological and psychological foundation for emotional regulation and compassionate engagement [8] and can be conceptualized as a personal resource within the JD‐R framework, whereas sleep deprivation can impair emotional regulation and reduce caring capacity [9].

Presenteeism, defined as working despite health problems, represents a job‐related demand that depletes energy and impairs functioning. Work loss associated with presenteeism reflects reduced productivity and impaired cognitive and emotional functioning at work [10]. When nurses work despite physical or psychological distress, their limited emotional resources may be directed toward self‐regulation, leaving fewer resources for compassionate care [11, 12].

Despite the dynamic and multifactorial nature of compassion competence, nursing research has predominantly relied on cross‐sectional designs and linear statistical approaches. Three research gaps remain. First, there is a temporal gap: cross‐sectional studies cannot examine long‐term changes in compassion competence. Second, there is a methodological gap: conventional linear models may not capture complex nonlinear, interactive, or threshold relationships among job demands, job resources, and personal resources, particularly when such relationships are not prespecified. Third, there is an explainable artificial intelligence (XAI) gap, reflected in the limited application of XAI methods to professional competencies in nursing workforce research.

To address these gaps, the present study combines 5‐year longitudinal data with an explainable machine learning approach. Ensemble tree‐based algorithms paired with SHapley Additive exPlanations (SHAP), recently applied to examine nurses’ professional quality of life [13], were used alongside conventional regression to examine potential nonlinear associations and model‐based feature contributions related to 5‐year changes in compassion competence.

## 2. Study Aims

This secondary analysis of 5‐year‐longitudinal data aimed to (i) identify 5‐year changes in nurses’ sleep quality, work loss due to presenteeism (hereafter, work loss), nursing work environment, and compassion competence; (ii) examine bivariate associations between changes in these factors and change in compassion competence; (iii) examine multivariable linear associations using multiple linear regression; and (iv) explore nonlinear associations and model‐based feature contributions using Random Forest, eXtreme Gradient Boosting (XGBoost), and Light Gradient Boosting Machine (LightGBM) combined with SHAP.

## 3. Methods

### 3.1. Study Design

This study was a secondary analysis of data from a prospective national longitudinal cohort of Korean clinical nurses, using a longitudinal observational design to examine factors associated with 5‐year changes in compassion competence. It is reported in accordance with the Strengthening the Reporting of Observational Studies in Epidemiology (STROBE) guideline.

Variables were examined within the JD‐R framework. Dimensions of the nursing work environment were considered job resources, work loss was considered a job‐related demand, and sleep quality was considered a personal health‐related resource. Demographic characteristics were included as covariates.

Multiple linear regression and XAI algorithms paired with TreeSHAP were applied as complementary approaches to examine linear and potentially nonlinear associations.

### 3.2. Participants

Longitudinal data were drawn from the national track of the Korean Nurses’ Compassion Competence Cohort (Compassion Cohort), a prospective cohort in which clinical nurses working in hospitals across South Korea were surveyed annually from 2018 to 2023 (T1–T6). Eligible nurses were registered nurses providing direct patient care in acute general or long‐term care hospitals with more than 100 beds and with at least three months of clinical experience. In the September 2026 integrated cohort database, deduplicated using unique participant identifiers, 2439 nurses were assigned to the national track, and 2035 completed the 2018 baseline survey (T1). The present analysis required eligible paired responses at both T1 (2018) and T5 (2022) and complete data for all study variables; participation at T2–T4 was not required. Of 348 nurses who met the paired response criterion, 16 were excluded because of incomplete data, leaving 332 nurses in the final analytic sample (Figure 1). Participants who did not meet these criteria were not uniformly classified as lost to follow‐up because patterns and reasons for nonparticipation varied across waves.

**FIGURE 1:**
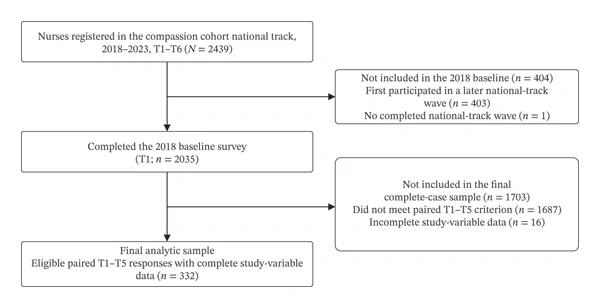
Flow of participants from the source cohort to the final analytic sample. Note: counts reflect the September 2026 integrated cohort database, deduplicated using unique participant identifiers. The national track of the compassion cohort is a prospective longitudinal cohort of Korean clinical nurses surveyed annually from 2018 to 2023 (T1–T6). Of 2439 unique nurses assigned to the national track, 404 did not complete the 2018 baseline survey: 403 first completed a national‐track questionnaire at a later wave and 1 completed no national‐track questionnaire wave. The remaining 2035 nurses completed the baseline survey (T1). Of these, 348 met the eligible paired T1–T5 response criterion; 16 had incomplete study‐variable data, leaving 332 nurses in the final analytic sample. Participation at T2–T4 was not required for the primary endpoint‐change analysis. The remaining 1703 baseline participants comprised 1687 who did not meet the paired T1–T5 response criterion and 16 with incomplete study‐variable data.

Age, sex, and clinical experience in the current unit were entered as baseline covariates using T1 values. Marital status and religious affiliation were categorized according to status transitions during the 5‐year follow‐up.

To assess potential selection bias, baseline characteristics were compared between the 332 included nurses and the 1703 baseline participants who were not included, using standardized mean differences (SMDs) based on the pooled standard deviation for continuous variables and on group proportions for categorical variables; an absolute SMD ≥ 0.10 was considered meaningful [14]. The results are presented in Supporting Table S1.

### 3.3. Measures

#### 3.3.1. Sleep Quality

Sleep quality was assessed using the Korean version [15] of the Pittsburgh Sleep Quality Index (PSQI; [16], which consists of 18 items in seven components scored from 0 to 3. Total scores range from 0 to 21, with higher scores indicating poorer sleep quality. Cronbach’s *α* was 0.77 at both T1 and T5.

#### 3.3.2. Work Loss due to Presenteeism

Work loss was measured using the Work Impairment Subscale of the Stanford Presenteeism Scale [17], translated into Korean by Lee and Jung [18]. Its 10 items are rated on a 5‐point Likert scale. For descriptive analyses, scores were converted to a 0–100 scale according to the developers’ guidelines; for the regression analysis, the item‐mean score (range 1–5) was used. Higher scores indicate greater work loss. Cronbach’s *α* was 0.59 at T1 and 0.76 at T5. The relatively low baseline reliability may have increased measurement error and attenuated or destabilized estimated associations involving work loss.

#### 3.3.3. Nursing Work Environment

The nursing work environment was measured using the Practice Environment Scale of the Nursing Work Index [7], validated in Korean by Cho et al. [19]. Its 29 items across five subdomains are rated on a 4‐point Likert scale, with higher scores indicating a more favorable environment. Cronbach’s *α* was 0.85 at T1 and 0.86 at T5.

#### 3.3.4. Compassion Competence

Compassion competence was measured using the 17‐item Compassion Competence Scale [1], which comprises communication, sensitivity, and insight domains rated on a 5‐point Likert scale. Mean scores were used, with higher scores indicating greater compassion competence. Cronbach’s *α* was 0.81 at T1 and 0.88 at T5.

### 3.4. Data Collection

Data were collected annually using online questionnaires and were anonymized before analysis. All measures included in the present analysis were administered at T1 (2018) and T5 (2022), whereas instrument coverage varied across the intermediate waves (e.g., the Practice Environment Scale of the Nursing Work Index was administered at T1, T4, and T5).

### 3.5. Data Analysis

Analyses were conducted using Python 3.13 with statsmodels 0.15.0, scikit‐learn 1.9.1, XGBoost 3.4.1, LightGBM 4.7.0, and SHAP 0.52.0.

#### 3.5.1. Preliminary Analyses

Descriptive statistics were calculated for all variables, and missing data patterns were examined before complete‐case selection. The primary analyses included participants with complete data for the study variables at T1 and T5.

#### 3.5.2. Longitudinal Change Analysis

Change scores (Δ) were calculated by subtracting T1 from T5 values. Paired *t*‐tests assessed differences between T1 and T5, and Pearson’s correlation coefficients examined associations between changes in the independent variables and change in compassion competence.

The primary estimand was the net 5‐year change from baseline to the final follow‐up; accordingly, T5−T1 change scores were used consistently as the outcome in the regression and machine learning analyses. This endpoint‐change approach quantified overall change but did not characterize within‐person trajectories across the intermediate T2–T4 assessments. As sensitivity analyses using all available waves, a linear mixed‐effects growth model with random intercepts and slopes was fitted to compassion competence from T1 to T5 (time in years), and a second model decomposed sleep quality, work loss, and the five work environment subdomains (available at T1, T4, and T5) into within‐person and between‐person components, adjusting for baseline age, sex, and clinical experience; both models were estimated using restricted maximum likelihood (Supporting Table S2). Change scores were retained rather than a baseline‐adjusted analysis of covariance because, in nonrandomized data, adjustment for baseline outcome values may yield biased estimates when predictors are associated with baseline levels [20].

#### 3.5.3. Multiple Linear Regression

Multiple linear regression with simultaneous entry (Enter method) of all 12 prespecified predictors was performed, without automated variable selection or stepwise elimination.

Normality of residuals was assessed using Q–Q plots and the Shapiro–Wilk test, homoscedasticity using standardized residual plots, independence of errors using the Durbin–Watson statistic, and multicollinearity using the variance inflation factor (VIF; values < 10 considered acceptable).

Unstandardized (*B*) and standardized (*β*) coefficients, 95% confidence intervals (CIs), *R* ^2^, and adjusted *R* ^2^ were reported. Standardized coefficients were calculated as *B* × SD(*X*)/SD(*Y*); in the regression, clinical experience in the current unit was entered in months and work loss on its item‐mean scale (range 1–5). As a sensitivity analysis, a baseline‐adjusted analysis of covariance additionally including T1 compassion competence was conducted (Supporting Table S3).

#### 3.5.4. Machine Learning Modeling

Three ensemble tree‐based models—Random Forest [21], XGBoost [22], and LightGBM [23]—were used to explore nonlinear associations and potential interactions without prespecifying functional forms. Ensemble methods reduce variance relative to single decision trees, and all models used the same predictors to ensure comparability.

#### 3.5.5. Model Training, Validation, and Evaluation

Marital status and religious affiliation were dummy‐coded using Category 1 as the reference (remained married and maintained religious affiliation, respectively), yielding 16 predictor terms for the 12 prespecified predictors; the same predictor set was used for linear regression and all ensemble models. To limit model complexity and the risk of overfitting, the maximum tree depth was set to 3 for XGBoost and LightGBM and to 5 for Random Forest; all ensemble models used 100 trees, and the learning rate for XGBoost and LightGBM was fixed at 0.05. These settings were held constant across folds, without grid search or early stopping; other hyperparameters were set to library defaults, and random_state = 42 was specified for all ensemble models.

Performance was evaluated using 100 repetitions of 5‐fold cross‐validation with identical splits across algorithms (random_state = 42), yielding 500 validation evaluations per algorithm (training sets, 265–266 participants; validation folds, 66–67 participants). Performance metrics were the coefficient of determination (*R* ^2^), root mean square error (RMSE), and mean absolute error (MAE), summarized as means ± standard deviations across folds. Because observations were reused across repetitions, these evaluations were not independent, and the standard deviations describe variability across partitions rather than sampling uncertainty. Full‐dataset performance, obtained by fitting and evaluating each model on all 332 participants, was reported separately as in‐sample fit rather than as evidence of predictive generalizability.

#### 3.5.6. XAI and SHAP Analysis

SHAP values were computed using the TreeSHAP algorithm [24] to quantify model‐based feature attributions for the ensemble models fitted to the full analytic sample.

Global feature importance was summarized as each predictor term’s mean absolute SHAP value, expressed as a percentage of the sum across all 16 predictor terms. Because signed SHAP values largely cancel out when averaged, mean signed SHAP values were not used to infer direction; instead, direction was summarized as the sign of the Spearman correlation between feature values and SHAP values (positive: higher feature values were associated with higher model predictions), with |*ρ*| < 0.10 regarded as indicating no consistent direction. To assess stability, TreeSHAP values were recomputed for each of the 500 fold‐specific training models, and the proportion of models in which each feature ranked among the five largest contributors and the proportion in which its direction matched that of the full‐sample model were recorded (Supporting Table S4).

SHAP values describe statistical associations within the fitted models rather than causal mechanisms and were interpreted as exploratory model‐based attributions.

### 3.6. Ethical Considerations

Written informed consent was obtained from all participants after the purpose and procedures of the study were explained. Data were anonymized prior to analysis. This study was approved by the Institutional Review Board of the affiliated institution (approval numbers: AJIRB‐SBR‐SUR‐17‐386 for Cohort 1.0 and AJIRB‐SBR‐SUR‐20‐328 for Cohort 2.0).

## 4. Results

### 4.1. General Characteristics of Participants

Table 1 presents participants’ baseline characteristics and changes in marital status and religious affiliation during follow‐up. The cohort consisted predominantly of young female nurses in the early stages of their clinical careers. Of the 332 nurses, 87 (26.2%) participated in all five waves from T1 to T5, 159 (47.9%) in four waves, and 86 (25.9%) in three waves. Compared with the 1703 nonincluded baseline participants (Supporting Table S1), included nurses were slightly younger (29.14 ± 4.26 vs. 29.71 ± 4.98 years; |SMD| = 0.12) and included a lower proportion of men (3.0% vs. 5.1%; |SMD| = 0.11), whereas mean clinical experience in the current unit was similar (|SMD| = 0.05), although fewer included nurses had less than 2 years of experience in the current unit (41.9% vs. 49.4%; |SMD| = 0.15). Although these differences exceeded the prespecified threshold, their absolute magnitudes were small; nevertheless, some selection bias cannot be ruled out.

**TABLE 1:** Detailed demographic characteristics (*n* = 332).

Variables		Categories	Mean ± SD or N (%)	Range
Time‐invariant (T1)	Age (years)		29.14 ± 4.26	20–60
		20–29	214 (64.5%)	
		30–39	110 (33.1%)	
		≥ 40	8 (2.4%)	
	Sex	Male	10 (3.0%)	
		Female	322 (97.0%)	
	Clinical experience in current unit (years)		2.55 ± 2.15	
		< 2	139 (41.9%)	
		2–< 4	126 (38.0%)	
		≥ 4	67 (20.2%)	

Time‐varying (T1–T5)	Marital status	Remained single	165 (49.7%)	
		Remained married	73 (22.0%)	
		Married during follow‐up	84 (25.3%)	
		Divorced during follow‐up	10 (3.0%)	
	Religious affiliation	No religion throughout	184 (55.4%)	
		Maintained religion	112 (33.7%)	
		Became religious	14 (4.2%)	
		No longer religious	22 (6.6%)	

Abbreviation: SD, standard deviation.

At baseline, the mean age was 29.14 years (SD = 4.26; range, 20–60), and 64.5% of participants were aged 20–29 years. Most participants were female (97.0%), and the mean clinical experience in the current unit was 2.55 years (SD = 2.15).

During follow‐up, approximately half of the participants remained single (49.7%), whereas 22.0% remained married, 25.3% became married, and 3.0% became divorced. More than half reported no religion throughout (55.4%), 33.7% maintained a religious affiliation, 4.2% became religious, and 6.6% were no longer religious.

### 4.2. Descriptive Statistics and Longitudinal Changes in Major Variables

Table 2 presents descriptive statistics and 5‐year changes (Δ = T5 − T1). Sleep quality scores decreased from 7.38 (SD = 3.76) to 6.91 (SD = 3.35) (mean change = −0.47; *p* = 0.010), indicating better sleep quality, whereas work loss scores increased from 58.16 (SD = 17.86) to 63.29 (SD = 14.36) (mean change = 5.13; *p* < 0.001).

**TABLE 2:** Descriptive statistics and longitudinal changes in the major study variables.

Characteristics	Range	Year 1	Year 5	Δ (Year 5**–**Year 1)	*p*
		Mean ± SD	Mean ± SD	Mean ± SD	
Sleep quality	0–21	7.38 ± 3.76	6.91 ± 3.35	−0.47 ± 3.27	0.010
Work loss	0–100	58.16 ± 17.86	63.29 ± 14.36	5.13 ± 18.67	< 0.001
Nursing work environment					
Nurse participation in hospital affairs	1–4	2.12 ± 0.55	2.21 ± 0.54	0.09 ± 0.60	0.008
Nursing foundations for quality of care	1–4	2.40 ± 0.57	2.48 ± 0.48	0.08 ± 0.58	0.011
Nurse manager ability, leadership, support	1–4	2.37 ± 0.70	2.52 ± 0.62	0.15 ± 0.80	< 0.001
Staffing and resource adequacy	1–4	1.93 ± 0.60	2.30 ± 0.57	0.37 ± 0.71	< 0.001
Collegial nurse–physician relations	1–4	2.44 ± 0.63	2.65 ± 0.55	0.22 ± 0.68	< 0.001
Compassion competence	1–5	3.68 ± 0.39	3.77 ± 0.45	0.09 ± 0.42	< 0.001

Abbreviation: SD, standard deviation.

All five nursing work environment subdomains increased significantly (*p* = 0.011 to *p* < 0.001), with the largest increase in staffing and resource adequacy (mean change = 0.37, SD = 0.71) and the smallest in nursing foundations for quality of care (mean change = 0.08, SD = 0.58).

Compassion competence increased from 3.68 (SD = 0.39) to 3.77 (SD = 0.45) (mean change = 0.09, SD = 0.42; *p* < 0.001; Cohen’s dz = 0.21). In the growth model using all available waves (332 nurses; 1314 observations), compassion competence increased by 0.023 points per year (95% CI, 0.013–0.034; *p* < 0.001). In the model decomposing time‐varying predictors, none of the within‐person or between‐person work environment components was statistically significant, whereas higher person‐level average work loss was associated with higher compassion competence (Supporting Table S2).

### 4.3. Correlations Among Changes in Major Variables

Table 3 presents the bivariate correlations. Change in compassion competence was positively correlated with changes in nurse participation in hospital affairs (*r* = 0.202, *p* < 0.001), nursing foundations for quality of care (*r* = 0.190, *p* < 0.001), and collegial nurse–physician relations (*r* = 0.176, *p* < 0.001), but not with changes in nurse manager ability, leadership, and support (*r* = 0.103, *p* = 0.061) or staffing and resource adequacy (*r* = 0.059, *p* = 0.287).

**TABLE 3:** Correlations among baseline characteristics and 5‐year changes in the major study variables (*N* = 332).

Variables	1	2	3	4	5	6	7	8	9	10	11	12	13	14	15	16	17	18	19
1. Age (T1)	—																		
2. Sex	−0.076 (0.169)	—																	
3. MS_1 Remained married	0.424 (< 0.001)	−0.135 (0.014)	—																
4. MS_2 Remained single	−0.285 (< 0.001)	0.017 (0.764)	−0.525 (< 0.001)	—															
5. MS_3 Married during follow‐up	−0.116 (0.034)	0.098 (0.075)	−0.311 (< 0.001)	−0.580 (< 0.001)	—														
6. MS_4 Divorced during follow‐up	0.102 (0.063)	0.029 (0.593)	−0.094 (0.089)	−0.174 (0.001)	−0.103 (0.060)	—													
7. RA_1 Maintained religion	0.152 (0.005)	−0.038 (0.492)	0.083 (0.133)	−0.004 (0.940)	−0.068 (0.215)	−0.014 (0.800)	—												
8. RA_2 No religion throughout	−0.126 (0.022)	0.074 (0.178)	−0.051 (0.358)	−0.023 (0.677)	0.054 (0.326)	0.052 (0.348)	−0.796 (< 0.001)	—											
9. RA_3 Became religious	0.046 (0.403)	0.035 (0.525)	0.106 (0.054)	−0.057 (0.297)	−0.020 (0.716)	−0.037 (0.502)	−0.150 (0.006)	−0.234 (< 0.001)	—										
10. RA_4 No longer religious	−0.074 (0.176)	−0.105 (0.057)	−0.141 (0.010)	0.100 (0.069)	0.038 (0.491)	−0.047 (0.394)	−0.190 (< 0.001)	−0.297 (< 0.001)	−0.056 (0.310)	—									
11. Clinical experience (T1)	0.224 (< 0.001)	0.008 (0.885)	0.201 (< 0.001)	−0.198 (< 0.001)	0.020 (0.711)	0.039 (0.477)	0.021 (0.709)	0.022 (0.692)	−0.038 (0.494)	−0.052 (0.343)	—								
12. ΔSleep quality	0.056 (0.309)	−0.046 (0.405)	0.083 (0.133)	0.053 (0.339)	−0.110 (0.045)	−0.072 (0.188)	0.039 (0.481)	0.001 (0.982)	−0.035 (0.526)	−0.048 (0.383)	0.073 (0.186)	—							
13. ΔWork loss	−0.077 (0.160)	0.131 (0.017)	−0.103 (0.060)	0.019 (0.734)	0.040 (0.466)	0.093 (0.092)	−0.012 (0.831)	0.038 (0.492)	−0.010 (0.852)	−0.045 (0.413)	−0.118 (0.032)	−0.236 (< 0.001)	—						
14. ΔNPHA	0.087 (0.114)	0.045 (0.414)	0.096 (0.082)	−0.043 (0.432)	−0.050 (0.362)	0.023 (0.676)	0.070 (0.205)	−0.049 (0.374)	0.014 (0.805)	−0.046 (0.407)	0.008 (0.880)	0.065 (0.241)	0.066 (0.229)	—					
15. ΔNFQC	0.084 (0.128)	0.023 (0.673)	0.077 (0.159)	−0.017 (0.759)	−0.094 (0.088)	0.101 (0.066)	0.061 (0.265)	−0.023 (0.671)	−0.015 (0.788)	−0.058 (0.291)	0.030 (0.592)	0.092 (0.093)	0.132 (0.016)	0.721 (< 0.001)	—				
16. ΔNMALS	0.063 (0.254)	0.096 (0.081)	0.076 (0.170)	−0.066 (0.231)	−0.011 (0.837)	0.039 (0.482)	0.035 (0.523)	0.000 (0.996)	0.078 (0.156)	−0.131 (0.017)	0.070 (0.204)	−0.039 (0.481)	0.160 (0.003)	0.587 (< 0.001)	0.435 (< 0.001)	—			
17. ΔSRA	−0.010 (0.862)	0.022 (0.691)	0.002 (0.978)	0.026 (0.633)	−0.054 (0.329)	0.057 (0.304)	0.024 (0.665)	0.017 (0.764)	−0.025 (0.648)	−0.058 (0.292)	−0.065 (0.234)	0.032 (0.561)	0.140 (0.010)	0.570 (< 0.001)	0.478 (< 0.001)	0.398 (< 0.001)	—		
18. ΔCNPR	0.029 (0.592)	0.098 (0.076)	0.001 (0.980)	−0.005 (0.926)	0.002 (0.965)	0.005 (0.923)	0.080 (0.148)	−0.070 (0.205)	0.022 (0.683)	−0.030 (0.587)	−0.009 (0.874)	0.030 (0.587)	0.141 (0.010)	0.549 (< 0.001)	0.425 (< 0.001)	0.479 (< 0.001)	0.400 (< 0.001)	—	
19. ΔCC	−0.054 (0.328)	0.085 (0.123)	0.046 (0.409)	−0.094 (0.086)	0.089 (0.104)	−0.062 (0.258)	0.044 (0.423)	−0.038 (0.492)	0.054 (0.329)	−0.052 (0.348)	−0.041 (0.455)	0.056 (0.305)	0.013 (0.809)	0.202 (< 0.001)	0.190 (< 0.001)	0.103 (0.061)	0.059 (0.287)	0.176 (0.001)	—

*Note:* Values are Pearson correlation coefficients with two‐tailed *p* values in parentheses. Δ denotes T5 − T1 change scores; age, sex, and clinical experience in the current unit were measured at T1. MS and RA categories are dummy indicators; correlations among categories of the same variable are structurally negative. NPHA, nurse participation in hospital affairs; NFQC, nursing foundations for quality of care; NMALS, nurse manager ability, leadership, and support of nurses; SRA, staffing and resource adequacy.

Abbreviations: CC, compassion competence; CNPR, collegial nurse–physician relations; MS, marital status; RA, religious affiliation.

Change in compassion competence was not significantly correlated with baseline age (*r* = −0.054, *p* = 0.328), sex (*r* = 0.085, *p* = 0.123), clinical experience in the current unit (*r* = −0.041, *p* = 0.455), any marital status or religious affiliation category (|r| ≤ 0.094, *p* ≥ 0.086), or changes in sleep quality (*r* = 0.056, *p* = 0.305) or work loss (*r* = 0.013, *p* = 0.809).

The five work environment subdomains were positively intercorrelated (*r* = 0.398–0.721), most strongly between nurse participation in hospital affairs and nursing foundations for quality of care (*r* = 0.721, *p* < 0.001).

### 4.4. Multiple Linear Regression Analysis of Changes in Compassion Competence

Table 4 presents the results of the simultaneous‐entry multiple linear regression. All 12 prespecified predictors were retained, with marital status and religious affiliation each represented by three dummy variables (16 predictor terms). VIFs ranged from 1.07 to 3.10, the Durbin–Watson statistic was 1.932, and the residuals did not deviate from normality (Shapiro–Wilk *p* = 0.379).

**TABLE 4:** Multiple linear regression of 5‐year change in compassion competence (*N* = 332).

	Estimate (*B*)	Std. error	95% CI for *B*	Beta	*t (F)*	*p*	VIF	*R* ^2^	Adjust *R* ^2^	*F*
(Constant)	0.181	0.353	[−0.514, 0.875]	—	0.512	0.609	—	0.1034	0.0579	*F* (16, 315) = 2.27, *p* = 0.004
Age	−0.012	0.006	[−0.024, −0.0002]	−0.122	−2.00	0.046	1.31			
Sex	0.177	0.142	[−0.103, 0.456]	0.069	1.242	0.215	1.07			
Marital status	—	—	—	—	(2.273)	0.080	—			
MS_1	Ref.	—	—	—	—	—	—			
MS_2	−0.109	0.065	[−0.238, 0.020]	−0.131	−1.665	0.097	2.15			
MS_3	0.010	0.072	[−0.130, 0.151]	0.011	0.146	0.884	1.96			
MS_4	−0.188	0.140	[−0.464, 0.087]	−0.077	−1.343	0.180	1.15			
Religious affiliation	—	—	—	—	(0.549)	0.649	—			
RA_1	Ref.	—	—	—	—	—	—			
RA_2	−0.036	0.050	[−0.134, 0.061]	−0.043	−0.732	0.465	1.22			
RA_3	0.065	0.117	[−0.164, 0.295]	0.031	0.559	0.577	1.10			
RA_4	−0.085	0.098	[−0.277, 0.107]	−0.051	−0.871	0.384	1.18			
Clinical experience	−0.001	0.001	[−0.003, 0.001]	−0.054	−0.953	0.341	1.12			
Sleep quality	0.006	0.007	[−0.008, 0.020]	0.046	0.824	0.411	1.11			
Work loss	−0.004	0.026	[−0.054, 0.047]	−0.008	−0.138	0.890	1.17			
NPHA	0.101	0.065	[−0.027, 0.229]	0.146	1.547	0.123	3.10			
NFQC	0.090	0.057	[−0.023, 0.203]	0.125	1.574	0.116	2.20			
NMALS	−0.026	0.037	[−0.098, 0.047]	−0.049	−0.694	0.488	1.71			
SRA	−0.061	0.039	[−0.138, 0.016]	−0.104	−1.560	0.120	1.57			
CNPR	0.061	0.041	[−0.019, 0.142]	0.100	1.496	0.136	1.57			

*F* (16, 315) = 2.27, *p* = 0.004										

*Note: N* = 332. The dependent variable was change in compassion competence (T5 − T1). *B*, unstandardized coefficient; SE, standard error; Beta, standardized coefficient; Ref. = reference category. Marital status (MS) and religious affiliation (RA) were dummy‐coded using category 1 as the reference. MS_1 and RA_1 identify reference categories and were not entered as separate predictor terms. Coefficients for categories 2–4 represent adjusted differences from the corresponding reference category. Statistics in parentheses are joint F statistics for the three dummy coefficients of each categorical variable, with degrees of freedom (3, 315); other statistics are t statistics. Dashes indicate values that are not applicable or are not reported for a categorical variable as a whole. Beta was calculated as *B* × SD(*X*)/SD(*Y*). Clinical experience in the current unit was entered in months and work loss on its item‐mean scale (range 1–5); sleep quality, work loss, and work environment variables are T5 − T1 change scores. NPHA, nurse participation in hospital affairs; NFQC, nursing foundations for quality of care; NMALS, nurse manager ability, leadership, and support of nurses; SRA, staffing and resource adequacy.

Abbreviations: CI, confidence interval; CNPR, collegial nurse–physician relations; VIF, variance inflation factor.

The model was statistically significant (*F* (16, 315) = 2.27, *p* = 0.004) and explained 10.34% of the variance in change in compassion competence (*R* ^2^ = 0.1034; adjusted *R* ^2^ = 0.0579). Baseline age was the only significant predictor term (*B* = −0.012, SE = 0.006, *β* = −0.122, *p* = 0.046). Joint tests were not significant for marital status (*F* (3, 315) = 2.273, *p* = 0.080) or religious affiliation (*F* (3, 315) = 0.549, *p* = 0.649). Among work environment variables, nurse participation in hospital affairs had the largest positive standardized coefficient (*B* = 0.101, SE = 0.065, *β* = 0.146, *p* = 0.123), whereas staffing and resource adequacy had a negative coefficient (*B* = −0.061, SE = 0.039, *β* = −0.104, *p* = 0.120); neither was statistically significant. In the baseline‐adjusted sensitivity analysis (Supporting Table S3), T1 compassion competence was negatively associated with change (*B* = −0.386, *p* < 0.001), and none of the work environment change terms were statistically significant.

### 4.5. Model Performance and Validation

Table 5 compares model performance on the full dataset and in 100 repetitions of 5‐fold cross‐validation (500 validation evaluations per algorithm), using the same predictors for all models.

**TABLE 5:** Model performance comparison on the full dataset and in 100 repetitions of 5‐fold cross‐validation across algorithms.

Metric	Evaluation mode	Linear regression	XGBoost	Random forest	LightGBM
*R* ^2^	Full Dataset	0.1034 (10.34%)	0.4086 (40.86%)	0.3479 (34.79%)	0.3342 (33.42%)
	100‐Iteration 5‐Fold CV	−0.0337 ± 0.1005	−0.1283 ± 0.0959	−0.0691 ± 0.0682	−0.1143 ± 0.0959

RMSE	Full Dataset	0.3951	0.3209	0.3370	0.3405
	100‐Iteration 5‐Fold CV	0.4189 ± 0.0385	0.4378 ± 0.0397	0.4266 ± 0.0395	0.4350 ± 0.0389

MAE	Full Dataset	0.3076	0.2492	0.2657	0.2608
	100‐Iteration 5‐Fold CV	0.3270 ± 0.0305	0.3406 ± 0.0320	0.3306 ± 0.0315	0.3362 ± 0.0311

On the full dataset, all ensemble models yielded higher *R* ^2^ values than linear regression (*R* ^2^ = 0.1034, RMSE = 0.3951, MAE = 0.3076), with XGBoost showing the highest fit (*R* ^2^ = 0.4086, RMSE = 0.3209, MAE = 0.2492), followed by Random Forest (*R* ^2^ = 0.3479) and LightGBM (*R* ^2^ = 0.3342).

In cross‐validation, mean *R* ^2^ values were negative for all models (linear regression, −0.0337 ± 0.1005; XGBoost, −0.1283 ± 0.0959; Random Forest, −0.0691 ± 0.0682; LightGBM, −0.1143 ± 0.0959). Linear regression had the lowest mean RMSE (0.4189 ± 0.0385) and MAE (0.3270 ± 0.0305), followed by Random Forest, LightGBM, and XGBoost.

### 4.6. Nonlinear SHAP‐Based Machine Learning Analysis

SHAP values were computed using TreeSHAP for the ensemble models fitted to the full dataset. Baseline age, sex, and clinical experience in the current unit; transitions in marital status and religious affiliation; and 5‐year change scores for sleep quality, work loss, and the five work environment subdomains were included as features.

Mean absolute SHAP values identified a consistent set of prominent features across models (Figures 2, 3, and 4; Table 6). The sum of the absolute mean signed SHAP values corresponded to only 3.1%–4.5% of the sum of the mean absolute SHAP values, confirming that signed values largely cancel out when averaged.

**FIGURE 2:**
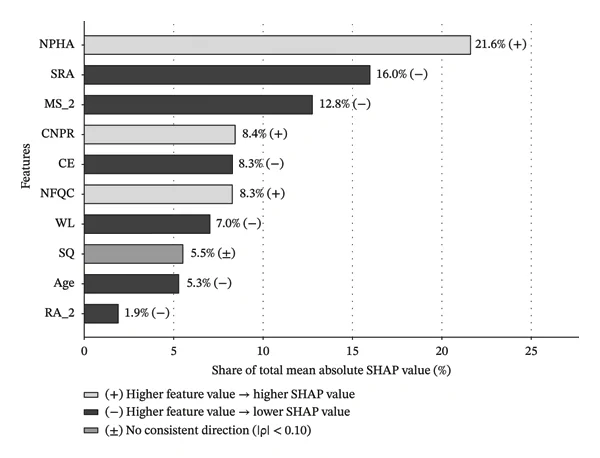
Mean absolute SHAP contributions from the XGBoost model (full dataset, *N* = 332). Note: percentages were calculated as 100x the mean absolute SHAP value of each predictor term divided by the sum of mean absolute SHAP values across all 16 predictor terms. The 10 largest contributions are shown; omitted terms remained in the model and in the denominator. Direction indicates the sign of the Spearman correlation between feature values and SHAP values: (+) higher feature values were associated with higher predicted change in compassion competence, (−) with lower predicted change, and (±) no consistent direction (|*ρ*| < 0.10). Values do not represent shares of explained variance, statistical significance, or causal effects. NPHA, NFQC, SRA, CNPR, WL, and SQ denote T5 − T1 change scores. MS_k and RA_k denote dummy indicators for category k of marital status and religious affiliation (reference: remained married and maintained religious affiliation, respectively); MS_2, remained single; MS_3, married during follow‐up; RA_2, no religion throughout. SHAP, SHapley Additive exPlanations; NPHA, nurse participation in hospital affairs; SRA, staffing and resource adequacy; CE, clinical experience in the current unit; NFQC, nursing foundations for quality of care. Abbreviations: WL, work loss; SQ, sleep quality; CNPR, collegial nurse–physician relations.

**FIGURE 3:**
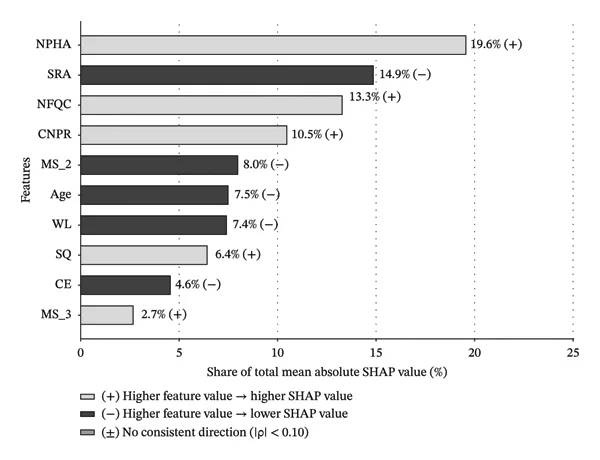
Mean absolute SHAP contributions from the Random Forest model (full dataset, *N* = 332). Note: Percentages were calculated as 100x the mean absolute SHAP value of each predictor term divided by the sum of mean absolute SHAP values across all 16 predictor terms. The 10 largest contributions are shown; omitted terms remained in the model and in the denominator. Direction indicates the sign of the Spearman correlation between feature values and SHAP values: (+) higher feature values were associated with higher predicted change in compassion competence, (−) with lower predicted change, and (±) no consistent direction (|*ρ*| < 0.10). Values do not represent shares of explained variance, statistical significance, or causal effects. NPHA, NFQC, SRA, CNPR, WL, and SQ denote T5 − T1 change scores. MS_k and RA_k denote dummy indicators for category k of marital status and religious affiliation (reference: remained married and maintained religious affiliation, respectively); MS_2, remained single; MS_3, married during follow‐up; RA_2, no religion throughout. SHAP, SHapley Additive exPlanations; NPHA, nurse participation in hospital affairs; SRA, staffing and resource adequacy; NFQC, nursing foundations for quality of care; CE, clinical experience in the current unit. Abbreviations: WL, work loss; SQ, sleep quality; CNPR, collegial nurse–physician relations.

**FIGURE 4:**
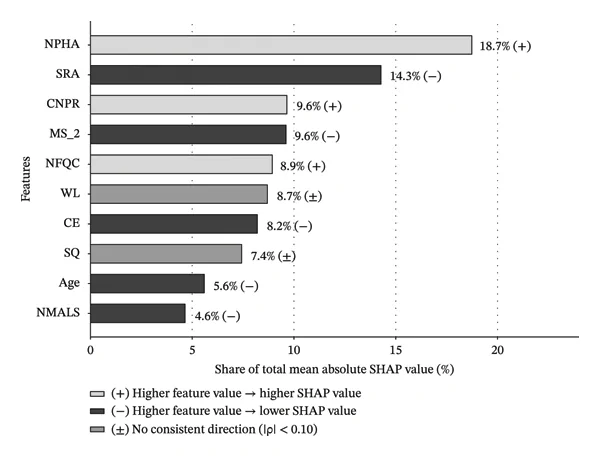
Mean absolute SHAP contributions from the LightGBM model (full dataset, *N* = 332). Note: Percentages were calculated as 100x the mean absolute SHAP value of each predictor term divided by the sum of mean absolute SHAP values across all 16 predictor terms. The 10 largest contributions are shown; omitted terms remained in the model and in the denominator. Direction indicates the sign of the Spearman correlation between feature values and SHAP values: (+) higher feature values were associated with higher predicted change in compassion competence, (−) with lower predicted change, and (±) no consistent direction (|*ρ*| < 0.10). Values do not represent shares of explained variance, statistical significance, or causal effects. NPHA, NFQC, NMALS, SRA, CNPR, WL, and SQ denote T5 − T1 change scores. MS_k and RA_k denote dummy indicators for category k of marital status and religious affiliation (reference: remained married and maintained religious affiliation, respectively); MS_2, remained single; MS_3, married during follow‐up; RA_2, no religion throughout. SHAP, SHapley Additive exPlanations; NPHA, nurse participation in hospital affairs; SRA, staffing and resource adequacy; NFQC, nursing foundations for quality of care; CE, clinical experience in the current unit; NMALS, nurse manager ability, leadership, and support of nurses. Abbreviations: CNPR, collegial nurse–physician relations; WL, work loss; SQ, sleep quality.

**TABLE 6:** Cross‐model comparison of SHAP contributions.

Rank	XGBoost	Random forest	LightGBM
1	NPHA 21.6% (+)	NPHA 19.6% (+)	NPHA 18.7% (+)
2	SRA 16.0% (−)	SRA 14.9% (−)	SRA 14.3% (−)
3	MS_2 12.8% (−)	NFQC 13.3% (+)	CNPR 9.6% (+)
4	CNPR 8.4% (+)	CNPR 10.5% (+)	MS_2 9.6% (−)
5	CE 8.3% (−)	MS_2 8.0% (−)	NFQC 8.9% (+)
6	NFQC 8.3% (+)	Age 7.5% (−)	WL 8.7% (±)
7	WL 7.0% (−)	WL 7.4% (−)	CE 8.2% (−)
8	SQ 5.5% (±)	SQ 6.4% (+)	SQ 7.4% (±)
9	Age 5.3% (−)	CE 4.6% (−)	Age 5.6% (−)
10	RA_2 1.9% (−)	MS_3 2.7% (+)	NMALS 4.6% (−)

*Note:* Values are the share of total mean absolute SHAP contribution for each model. Direction indicates the sign of the Spearman correlation between feature values and SHAP values: (+) positive, (−) negative, and (±) no consistent direction (|*ρ*| < 0.10). The 10 largest contributions are shown; all 16 predictor terms were retained in model fitting and in the denominator. SHAP values are model‐based attributions and should not be interpreted as causal effects. SHAP, SHapley Additive exPlanations; NPHA, nurse participation in hospital affairs; SRA, staffing and resource adequacy; CE, clinical experience in the current unit; NFQC, nursing foundations for quality of care; NMALS, nurse manager ability, leadership, and support.

Abbreviations: CNPR, collegial nurse–physician relations; MS, marital status; RA, religious affiliation; SQ, sleep quality; WL, work loss.

In XGBoost (Figure 2), change in nurse participation in hospital affairs (NPHA) had the largest share (21.6%; positive direction), followed by change in staffing and resource adequacy (SRA; 16.0%; negative), remaining single relative to remaining married (MS_2; 12.8%; negative), change in collegial nurse–physician relations (CNPR; 8.4%; positive), clinical experience in the current unit (CE; 8.3%; negative), and change in nursing foundations for quality of care (NFQC; 8.3%; positive).

In Random Forest (Figure 3), NPHA again had the largest share (19.6%; positive), followed by SRA (14.9%; negative), NFQC (13.3%; positive), CNPR (10.5%; positive), and MS_2 (8.0%; negative).

In LightGBM (Figure 4), NPHA (18.7%; positive) and SRA (14.3%; negative) again ranked first and second, followed by CNPR (9.6%; positive), MS_2 (9.6%; negative), and NFQC (8.9%; positive), whereas changes in work loss (8.7%) and sleep quality (7.4%) showed no consistent direction.

### 4.7. Integrated Synthesis Across Models

Table 6 summarizes the 10 largest mean absolute SHAP contributions and their directions for each model; all 16 predictor terms were retained in model fitting and in the denominator.

Changes in NPHA and SRA were the two most important features in all three models (combined share, 33.0%–37.6%), with consistently positive and negative directions, respectively. Changes in CNPR (8.4%–10.5%) and NFQC (8.3%–13.3%) also ranked among the six most important features in all models, both with positive directions. MS_2 (8.0%–12.8%), CE (4.6%–8.3%), and age (5.3%–7.5%) showed consistently negative directions.

These directions were concordant with the signs of the corresponding regression coefficients (Table 4), whereas changes in sleep quality and work loss had no consistent direction across models. In the stability analysis (Supporting Table S4), NPHA and SRA ranked among the five most important features in 92%–99% of the 500 fold‐specific models of each algorithm, with directions matching the full‐sample direction in all models. NFQC and CNPR ranked among the five most important features in 58%–88% of models, with direction agreement of 97%–100%. By contrast, the sign of the mean signed SHAP value agreed with the full‐sample sign in only 39%–71% of models for these four features.

Among the marital status and religious affiliation dummy terms, only MS_2 ranked among the most important features; each remaining dummy term accounted for less than 3.5% of the total in every model.

## 5. Discussion

This study examined 5‐year changes in nurses’ compassion competence and their associations with personal and job‐related factors within the JD‐R framework, integrating conventional regression with explainable machine learning.

Across the ensemble models, changes in nursing work environment features, particularly nurse participation in hospital affairs and staffing and resource adequacy, followed by collegial nurse–physician relations and nursing foundations for quality of care, were consistently the most prominent model‐based contributors, with directions that were stable across folds and concordant with the signs of the regression coefficients. However, because these associations were not statistically significant in the adjusted linear model and cross‐validated predictive performance was limited, these patterns should be interpreted as exploratory characteristics of the fitted models.

### 5.1. Five‐Year Changes in Compassion Competence

Compassion competence increased significantly over 5 years, although the magnitude was modest (a mean change of 0.09 points, or approximately 0.02 points per year in the growth model). This finding suggests that compassion competence is not a fixed dispositional trait but a dynamic professional competency that can develop through clinical experience, learning, and professional socialization [1]. Given that most participants were early career nurses, accumulating clinical experience and adaptation to professional roles may have contributed to this increase.

This interpretation is consistent with evidence that compassion‐related competencies can be strengthened through educational and experiential processes. For example, Ayed et al. [25] reported that high‐fidelity simulation enhanced nursing students’ self‐awareness, empathy, and patient‐centered care; because that study involved students and did not examine longitudinal change among practicing nurses, it provides indirect support for a learning‐based developmental process rather than direct evidence for the present findings. Reflective practices and supportive organizational contexts have also been highlighted as fostering compassionate care [4], and from a JD‐R perspective, job resources such as organizational support and professional development opportunities may help sustain the capacities required for compassionate practice.

### 5.2. Linear Versus Nonlinear Modeling

The comparison between linear regression and tree‐based models highlights the distinction between in‐sample fit and out‐of‐sample predictive performance. The regression model explained only 10.34% of the variance in 5‐year change in compassion competence, and only baseline age was statistically significant. This limited explanatory power indicates substantial unexplained variability, which may reflect unmeasured individual, occupational, or organizational factors; measurement error, including the lower baseline reliability of the work loss measure; and the variability inherent in change scores. The present analysis cannot distinguish among these sources, and limited linear explanatory power alone does not indicate nonlinear relationships.

The ensemble models achieved higher full‐sample *R* ^2^ values (0.3342–0.4086), but this advantage was not retained in repeated cross‐validation: mean cross‐validated *R* ^2^ values were negative for all models (Table 5), and linear regression showed the lowest mean cross‐validated RMSE and MAE. Thus, the higher in‐sample fit of the more flexible models did not translate into superior out‐of‐sample predictive performance.

These findings do not establish whether nonlinear relationships better characterize the underlying associations. Higher in‐sample fit may reflect the capacity of flexible models to capture sample‐specific variation, including noise, rather than reproducible nonlinear patterns; accordingly, the results do not support claims of validated threshold effects, interactions, or predictive relationships. TreeSHAP therefore provides only a complementary description of how the fitted models distribute predictions across variables, and SHAP‐based importance and direction should not be interpreted as effect sizes, statistical significance, or causal mechanisms. Notably, the importance rankings and directions of the leading work environment features were reproducible across data partitions, whereas mean signed SHAP values were not; the latter should therefore not be used to infer direction.

### 5.3. Role of the Nursing Work Environment

Nursing work environment variables featured prominently in the exploratory attribution profiles, although none of their coefficients reached statistical significance in the adjusted regression. Changes in nurse participation in hospital affairs, staffing and resource adequacy, collegial nurse–physician relations, and nursing foundations for quality of care had the largest mean absolute SHAP values in all ensemble models, whereas nurse manager ability, leadership, and support contributed little. Given the limited cross‐validated performance, this prominence should not be interpreted as evidence of independent effects or validated predictive importance.

The JD‐R framework provides a conceptual basis for viewing a supportive nursing work environment as a potential resource for compassionate practice [5]. Participation in organizational decision‐making, collaborative professional relationships, adequate staffing, and managerial support for quality care may help nurses sustain their capacity for compassionate engagement, consistent with literature linking nursing practice environments with nurses’ emotional and professional outcomes [6, 7], although the present analyses did not directly test these mechanisms.

Larger increases in nurse participation in hospital affairs, collegial nurse–physician relations, and nursing foundations for quality of care were associated with higher model predictions of change in compassion competence, whereas larger increases in staffing and resource adequacy were associated with lower predictions. The negative direction for staffing and resource adequacy contrasts with its positive, although nonsignificant, bivariate correlation with change in compassion competence (*r* = 0.059) and emerged only when the other work environment subdomains, with which it was moderately correlated (*r* = 0.40–0.57), were considered simultaneously. This pattern may therefore reflect statistical adjustment among correlated subdomains rather than a detrimental effect of staffing resources. Moreover, these directions describe associations within the fitted models and do not establish that changing any work environment feature would change compassion competence.

Overall, these findings identify work environment factors that warrant further investigation rather than establishing intervention targets or confirming specific JD‐R mechanisms. Theoretically, the present study extends the application of the JD‐R framework by modeling a professional competency as a longitudinal outcome and by examining job resources, job‐related demands, and personal health‐related resources simultaneously in relation to 5‐year change. Future longitudinal studies should account for baseline competence and relevant confounders and directly assess the proposed mechanisms.

### 5.4. Personal and Health‐Related Factors

Age and clinical experience in the current unit showed negative directions in all ensemble models, and baseline age was the only predictor term significantly associated with change in the adjusted regression (*B* = −0.012, *p* = 0.046). Because the outcome represents change rather than level, these findings suggest that younger and less experienced nurses tended to show larger increases, not that experienced nurses have lower compassion competence; the associations were small. Benner’s novice‐to‐expert framework [26] suggests that professional capabilities may develop rapidly in early career stages through diverse clinical experiences and professional socialization, with smaller gains from additional years of practice thereafter; from a JD‐R perspective, further development may also depend on resources such as continuing education and organizational support. However, this study did not test career‐stage trajectories or a plateau effect. Moreover, the baseline‐adjusted analysis showed larger increases among nurses with lower baseline compassion competence (Supporting Table S3), consistent with regression toward the mean or greater room for development; ceiling effects were not directly evaluated.

Remaining single throughout follow‐up (MS_2), relative to remaining married, showed a consistently negative direction across models and a negative but nonsignificant regression coefficient (*B* = −0.109, *p* = 0.097), whereas the other dummy terms contributed little. These category‐specific attributions should not be interpreted as evidence of protective effects of marriage or of social or spiritual resources, which were not directly evaluated.

Changes in work loss and sleep quality showed weak or inconsistent directions across ensemble models and were not associated with change in compassion competence in the regression; thus, the present data do not support a direct association between changes in these health‐related factors and change in compassion competence. In the growth model, higher person‐level average work loss was associated with higher compassion competence (Supporting Table S2), a finding that requires cautious interpretation and replication.

Personal resources nevertheless remain relevant within the JD‐R framework. In a cross‐sectional study of neonatal intensive care unit nurses, Ayed [27] reported an association between emotional intelligence and coping behaviors, suggesting that emotional resources may shape how nurses manage occupational demands. However, that study did not examine compassion competence, and neither emotional intelligence nor coping was measured in the present study. Future research should examine such personal resources and their interplay with the work environment, using clearly defined measures, repeated assessments, and independent validation.

### 5.5. Implications for Nursing Management

These findings suggest that compassion competence should be considered within both individual and organizational contexts rather than solely as an individual attribute. Participation in organizational decision‐making, nurse–physician collaboration, leadership support, and staffing may warrant further assessment in research on compassionate practice. However, because no work environment variable was statistically significant in the adjusted regression and cross‐validated predictive performance was limited, the findings do not establish whether modifying any specific organizational factor would improve compassion competence. Within these constraints, maintaining adequate nurse staffing and strengthening shared governance structures that support nurses’ participation in decision‐making could be evaluated as management approaches in future intervention studies.

XAI may complement nursing workforce research by describing model‐based contributions and complex patterns that are not evident in linear analyses. In the present study, however, attribution patterns should be used for hypothesis generation rather than for predictive decision‐making, intervention selection, or resource allocation, and future applications require replication and independent external validation.

### 5.6. Limitations

Several methodological limitations should be considered when interpreting these findings:

• Secondary Analysis and Residual Confounding: As a secondary analysis of an existing cohort dataset, variable selection and operational definitions were constrained by the design of the primary study. Consequently, potentially relevant factors that were not included in the original dataset, such as personality traits, psychological resilience, and hospital‐level structural changes, were not fully accounted for. Residual confounding may therefore remain.
• Selection Bias and Generalizability: Participants were recruited through nonprobability sampling and were required to have responded at both T1 and T5 with complete data, which may have introduced selection and attrition bias if nonincluded baseline participants differed systematically from those analyzed. Included nurses were slightly younger, included a lower proportion of men (|SMD| = 0.11–0.12), and less often had under 2 years of experience in the current unit than nonincluded baseline participants (Supporting Table S1); although these differences were small, the findings may be less applicable to older and newly assigned nurses. The sample consisted predominantly of female nurses (97.0%) working in hospitals in South Korea; therefore, caution is warranted when generalizing to male nurses, other healthcare settings, or countries with different healthcare systems, staffing structures, organizational cultures, and interprofessional practice patterns. Future studies should include more diverse samples across sex, healthcare settings, institutions, and countries.
• Self‐Report Measures and Scale Reliability: All study variables were assessed using self‐report questionnaires, which may be susceptible to social desirability bias, recall bias, and common method variance. In addition, the relatively low internal consistency of the work loss measure at T1 (Cronbach’s *α* = 0.59) may have introduced measurement error; although reliability improved at T5 (*α* = 0.76), findings involving work loss should be interpreted with caution.
• Machine Learning Model Performance and Overfitting: Although model complexity was constrained and internal validation was performed using 100 repetitions of 5‐fold cross‐validation, the gap between full‐sample and cross‐validated performance indicates that sample‐specific overfitting cannot be fully excluded, and cross‐validated out‐of‐sample performance was limited. The SHAP findings should therefore be interpreted as exploratory, model‐based attributions rather than as evidence of robust predictive performance or causal relationships. Mean signed SHAP values were also unstable across data partitions (Supporting Table S4); therefore, importance was summarized using mean absolute SHAP values. Because repeated cross‐validation within a single cohort constitutes internal validation only, external validation using independent samples from other hospitals is required.
• Causal Inference and Longitudinal Modeling: Although the longitudinal data enabled the examination of change over 5 years, the observational design precludes causal inference, and the observed associations should not be interpreted as evidence that changes in specific personal or organizational factors caused changes in compassion competence. In addition, the primary analyses focused on 5‐year change scores (T5 − T1) rather than the full trajectory across T1–T5, so changes at intermediate time points may not have been fully captured. Although a growth model using all available waves supported the overall increase, intermediate assessments were incomplete for many participants, and the work environment was measured only at T1, T4, and T5. Future studies using prospective multisite samples and mixed‐effects models, growth‐curve models, or generalized estimating equations may better characterize within‐person change.

### 5.7. Conclusions

Drawing on longitudinal data from an annually surveyed cohort, this study observed an increase in nurses’ compassion competence from T1 to T5, suggesting that this professional competency may change over time. However, the factors underlying individual differences in this change remain unclear.

The linear regression model explained a limited proportion of the variance, and only baseline age reached statistical significance. Although the tree‐based ensemble models demonstrated better fit in the full sample, all models yielded negative mean cross‐validated *R* ^2^ values, indicating limited out‐of‐sample predictive performance. TreeSHAP analyses consistently identified changes in nursing work environment features, particularly nurse participation in hospital affairs and staffing and resource adequacy, as the most prominent contributors to the fitted models. However, these attribution patterns do not establish causal effects or specific targets for intervention.

The JD‐R framework provides a conceptual basis for further investigation of individual and organizational factors involved in the development of compassion competence, but the present findings do not establish whether modifying specific workplace resources would improve it. Prospective multisite studies incorporating additional predictors, repeated‐measures modeling, and independent external validation are needed to clarify these relationships and their implications for nursing management and policy.

## Funding

This work was supported by the National Research Foundation of Korea(NRF) grant funded by the Korea Government (MSIT) (NRF‐2019R1A2C1085924; RS‐2025‐16070957), by the Gyeonggi Regional Innovation System & Education Project (Gyeonggi RISE Project), funded by the Ministry of Education and Gyeonggi Province, and research fund of Ajou University Medical Center (2026).

## Conflicts of Interest

The authors declare no conflicts of interest.

## Supporting Information

Additional supporting information can be found online in the Supporting Information section.

## Supporting information


**Supporting Information 1** Supporting File 1 (Supporting_Tables_S1‐S4_REVISED.docx) contains four supporting tables: Table S1 compares baseline characteristics of nurses included in and not included in the final analytic sample; Table S2 presents linear mixed‐effects models of compassion competence using all available waves as a sensitivity analysis; Table S3 reports a baseline‐adjusted analysis of covariance of 5 year change in compassion competence; and Table S4 summarizes the importance and stability of SHAP contributions across 500 fold‐specific models from 100 repetitions of 5‐fold cross‐validation.

**Supporting Information 2** STROBE Statement—Checklist of items for cohort studies.

## Data Availability

The deidentified analytic dataset (*N* = 332), codebook, analysis code, and equivalent Stata 19 syntax for the conventional and mixed‐effects analyses are available from the corresponding author upon reasonable request, subject to cohort governance and institutional approval, because the cohort consent and ethics approval do not permit public deposition of individual‐level data.
