# Supplementary material for: Five‐Year Changes in Nurses’ Compassion Competence and Associated Factors: A Longitudinal Study Using Explainable Machine Learning
Source: J Nurs Manag. 2026 Oct 25;2026:3937997. doi: 10.1155/jonm/3937997 (PMC13695800; doi:10.1155/jonm/3937997)
Supplement: Supplementary file 1 [file JONM-2026-3937997-s001.docx]

**STROBE Statement — Checklist of items for cohort studies**

Five-Year Changes in Nurses’ Compassion Competence and Associated Factors: A Longitudinal Study Using Explainable Machine Learning

| **Section/Topic** | **Item** | **Recommendation (cohort study)** | **Reported in** |
| --- | --- | --- | --- |
| Title and abstract | 1 | (a) Indicate the study’s design with a commonly used term in the title or abstract; (b) provide an informative and balanced summary | Title; Abstract (Methods: secondary analysis of a national cohort) |
| **Introduction** | | | |
| Background/rationale | 2 | Explain the scientific background and rationale | Section 1 |
| Objectives | 3 | State specific objectives, including any prespecified hypotheses | Section 2 |
| **Methods** | | | |
| Study design | 4 | Present key elements of study design early in the paper | Section 3.1 |
| Setting | 5 | Describe the setting, locations, and relevant dates, including periods of recruitment, exposure, follow-up, and data collection | Sections 3.2 and 3.4 |
| Participants | 6 | (a) Eligibility criteria, sources and methods of selection, methods of follow-up | Section 3.2; Figure 1 |
| Variables | 7 | Clearly define all outcomes, exposures, predictors, potential confounders, and effect modifiers | Sections 3.1–3.3 |
| Data sources/measurement | 8 | Give sources of data and details of methods of assessment for each variable | Section 3.3 |
| Bias | 9 | Describe any efforts to address potential sources of bias | Sections 3.2 (SMD comparison), 3.5.5 (complexity constraints, cross-validation); Supplementary Table S1 |
| Study size | 10 | Explain how the study size was arrived at | Section 3.2; Figure 1 |
| Quantitative variables | 11 | Explain how quantitative variables were handled in the analyses | Sections 3.3, 3.5.2, 3.5.3, and 3.5.5 |
| Statistical methods | 12 | (a) Describe all statistical methods, including those used to control for confounding; (b) subgroups and interactions; (c) missing data; (d) loss to follow-up; (e) sensitivity analyses | (a) Sections 3.5.2–3.5.6; (b) Section 3.5.4; (c) Sections 3.2 and 3.5.1 (complete-case analysis); (d) Section 3.2; (e) Sections 3.5.2 and 3.5.3 (growth models, ANCOVA); Supplementary Tables S2–S4 |
| **Results** | | | |
| Participants | 13 | (a) Report numbers of individuals at each stage of the study; (b) give reasons for non-participation; (c) consider use of a flow diagram | Section 3.2; Section 4.1; Figure 1 |
| Descriptive data | 14 | (a) Characteristics of participants; (b) number of participants with missing data for each variable; (c) summarise follow-up time | (a) Table 1; Supplementary Table S1; (b) Figure 1, Supplementary Table S1 note; (c) Section 4.1 (number of waves) |
| Outcome data | 15 | Report numbers of outcome events or summary measures over time | Section 4.2; Table 2; Supplementary Table S2 |
| Main results | 16 | (a) Unadjusted and confounder-adjusted estimates and their precision (e.g., 95% CI); (b) category boundaries; (c) absolute risk (if relevant) | (a) Tables 3 and 4; Section 4.4; (b) Table 1; (c) not applicable |
| Other analyses | 17 | Report other analyses done (subgroups, interactions, sensitivity analyses) | Sections 4.2, 4.4–4.7; Tables 5 and 6; Figures 2–4; Supplementary Tables S2–S4 |
| **Discussion** | | | |
| Key results | 18 | Summarise key results with reference to study objectives | Section 5 (opening paragraphs) |
| Limitations | 19 | Discuss limitations, taking into account sources of potential bias or imprecision | Section 5.6 |
| Interpretation | 20 | Give a cautious overall interpretation considering objectives, limitations, multiplicity of analyses, and results from similar studies | Sections 5.1–5.5 and 5.7 |
| Generalisability | 21 | Discuss the generalisability (external validity) of the study results | Section 5.6 (Selection Bias and Generalizability) |
| **Other information** | | | |
| Funding | 22 | Give the source of funding and the role of the funders | Title page (Funding) |

Locations refer to section numbers of the anonymized manuscript. Checklist adapted from von Elm, E., Altman, D. G., Egger, M., Pocock, S. J., Gøtzsche, P. C., & Vandenbroucke, J. P. (2007). The Strengthening the Reporting of Observational Studies in Epidemiology (STROBE) statement: Guidelines for reporting observational studies. The Lancet, 370(9596), 1453–1457. https://doi.org/10.1016/S0140-6736(07)61602-X
