# Supplementary material for: Five‐Year Changes in Nurses’ Compassion Competence and Associated Factors: A Longitudinal Study Using Explainable Machine Learning
Source: J Nurs Manag. 2026 Oct 25;2026:3937997. doi: 10.1155/jonm/3937997 (PMC13695800; doi:10.1155/jonm/3937997)
Supplement: Supplementary file 2 [file JONM-2026-3937997-s002.docx]

**Supplementary**

Five-Year Changes in Nurses’ Compassion Competence and Associated Factors: A Longitudinal Study Using Explainable Machine Learning

Table S1. Baseline (T1, 2018) characteristics of nurses included in and not included in the final analytic sample

| **Variables** | **Categories** | **Included (n = 332)** | **Not included (n = 1,703)** | **\|SMD\|** |
| --- | --- | --- | --- | --- |
| Age (years) | Mean ± SD | 29.14 ± 4.26 | 29.71 ± 4.98 | 0.12 |
|  | 20–29 | 214 (64.5%) | 1009 (59.3%) | 0.11 |
|  | 30–39 | 110 (33.1%) | 608 (35.7%) | 0.05 |
|  | ≥ 40 | 8 (2.4%) | 84 (4.9%) | 0.13 |
| Sex | Male | 10 (3.0%) | 87 (5.1%) | 0.11 |
|  | Female | 322 (97.0%) | 1616 (94.9%) |  |
| Clinical experience in current unit (years) | Mean ± SD | 2.55 ± 2.15 | 2.43 ± 2.20 | 0.05 |
|  | < 2 | 139 (41.9%) | 840 (49.4%) | 0.15 |
|  | 2–< 4 | 126 (38.0%) | 513 (30.1%) | 0.17 |
|  | ≥ 4 | 67 (20.2%) | 349 (20.5%) | 0.01 |

SD, standard deviation; SMD, standardized mean difference. Among non-included participants, age was available for 1,701 and clinical experience for 1,702; percentages use nonmissing denominators. Age ranged from 20 to 60 years (included) and from 20 to 57 years (not included). SMDs for means were calculated using the pooled standard deviation (from unrounded data), and SMDs for categories compare the proportion in each category; for sex, a single SMD is shown for the binary variable. |SMD| ≥ 0.10 was considered meaningful.

**Table S2.** Linear mixed-effects models of compassion competence using all available waves (sensitivity analysis)

Panel A. Observed compassion competence by wave (analytic sample, N = 332)

| **Wave** | **n** | **Mean** | **SD** |
| --- | --- | --- | --- |
| T1 | 332 | 3.68 | 0.39 |
| T2 | 131 | 3.71 | 0.39 |
| T3 | 248 | 3.65 | 0.41 |
| T4 | 271 | 3.75 | 0.40 |
| T5 | 332 | 3.77 | 0.45 |

Panel B. Model 1: unconditional growth model (persons = 332; observations = 1314)

| **Fixed effect** | **B** | **SE** | **95% CI** | **p** |
| --- | --- | --- | --- | --- |
| Intercept | 3.667 | 0.020 | [3.627, 3.706] | <.001 |
| Time (years since T1) | 0.023 | 0.005 | [0.013, 0.034] | <.001 |

Random effects: intercept variance = 0.084; slope variance = 0.0023; intercept–slope covariance = 0.0003; residual variance = 0.068.

Panel C. Model 2: within- and between-person effects of time-varying predictors (waves T1, T4, T5; persons = 332; observations = 928)

| **Fixed effect** | **B** | **SE** | **95% CI** | **p** |
| --- | --- | --- | --- | --- |
| Intercept | 3.122 | 0.338 | [2.460, 3.785] | <.001 |
| Time (years since T1) | 0.018 | 0.006 | [0.006, 0.029] | .004 |
| NPHA, within-person | 0.017 | 0.049 | [−0.079, 0.114] | .724 |
| NPHA, between-person | 0.040 | 0.086 | [−0.129, 0.209] | .640 |
| NFQC, within-person | 0.048 | 0.043 | [−0.036, 0.132] | .261 |
| NFQC, between-person | 0.086 | 0.073 | [−0.058, 0.230] | .240 |
| NMALS, within-person | 0.038 | 0.028 | [−0.017, 0.092] | .178 |
| NMALS, between-person | −0.040 | 0.058 | [−0.153, 0.073] | .488 |
| SRA, within-person | −0.010 | 0.029 | [−0.066, 0.047] | .735 |
| SRA, between-person | −0.044 | 0.060 | [−0.162, 0.073] | .462 |
| CNPR, within-person | 0.025 | 0.030 | [−0.035, 0.084] | .412 |
| CNPR, between-person | 0.064 | 0.057 | [−0.048, 0.177] | .263 |
| Sleep quality, within-person | 0.004 | 0.005 | [−0.006, 0.014] | .456 |
| Sleep quality, between-person | 0.005 | 0.007 | [−0.008, 0.018] | .425 |
| Work loss (per 10 points), within-person | 0.010 | 0.009 | [−0.008, 0.028] | .275 |
| Work loss (per 10 points), between-person | 0.047 | 0.017 | [0.014, 0.079] | .005 |
| Age at T1 | −0.0001 | 0.005 | [−0.010, 0.009] | .951 |
| Sex (1 = male, 2 = female) | 0.012 | 0.115 | [−0.214, 0.238] | .919 |
| Clinical experience in current unit (years) | −0.016 | 0.009 | [−0.034, 0.002] | .073 |

*Note.* Models were estimated by restricted maximum likelihood with random intercepts and random slopes for time (unstructured covariance). In Model 2, within-person components are deviations from each nurse’s mean across waves and between-person components are the person means. NPHA, nurse participation in hospital affairs; NFQC, nursing foundations for quality of care; NMALS, nurse manager ability, leadership, and support of nurses; SRA, staffing and resource adequacy; CNPR, collegial nurse–physician relations. In Panel A, n denotes the number of nurses with compassion competence scores at each wave. Model 2 included observations with complete data on the time-varying predictors at T1, T4, and T5 (928 observations). In Model 2, clinical experience in the current unit was entered in years and work loss on the 0–100 scale (per 10 points).

**Table S3.** Baseline-adjusted analysis of covariance of five-year change in compassion competence (sensitivity analysis, N = 332)

| **Term** | **B** | **SE** | **95% CI** | **t** | **p** |
| --- | --- | --- | --- | --- | --- |
| (Constant) | 1.606 | 0.387 | [0.845, 2.368] | 4.150 | <.001 |
| Age | −0.0113 | 0.0056 | [−0.0222, −0.0003] | −2.021 | .044 |
| Sex | 0.196 | 0.135 | [−0.069, 0.460] | 1.453 | .147 |
| MS_2 (remained single) | −0.111 | 0.060 | [−0.230, 0.008] | −1.828 | .068 |
| MS_3 (married during follow-up) | −0.019 | 0.066 | [−0.150, 0.111] | −0.289 | .773 |
| MS_4 (divorced during follow-up) | −0.202 | 0.131 | [−0.459, 0.055] | −1.546 | .123 |
| RA_2 (no religion throughout) | −0.061 | 0.046 | [−0.153, 0.030] | −1.320 | .188 |
| RA_3 (became religious) | −0.003 | 0.109 | [−0.217, 0.212] | −0.024 | .981 |
| RA_4 (no longer religious) | −0.126 | 0.091 | [−0.305, 0.053] | −1.385 | .167 |
| Clinical experience in current unit (years) | −0.015 | 0.010 | [−0.035, 0.005] | −1.435 | .152 |
| Δ Sleep quality | 0.009 | 0.007 | [−0.005, 0.022] | 1.278 | .202 |
| Δ Work loss (0–100 scale) | −0.0007 | 0.0012 | [−0.0031, 0.0016] | −0.608 | .543 |
| Δ NPHA | 0.110 | 0.061 | [−0.010, 0.230] | 1.801 | .073 |
| Δ NFQC | 0.028 | 0.054 | [−0.079, 0.134] | 0.511 | .609 |
| Δ NMALS | −0.027 | 0.034 | [−0.095, 0.040] | −0.802 | .423 |
| Δ SRA | −0.059 | 0.037 | [−0.131, 0.013] | −1.603 | .110 |
| Δ CNPR | 0.054 | 0.038 | [−0.021, 0.130] | 1.425 | .155 |
| Compassion competence at T1 | −0.386 | 0.056 | [−0.497, −0.275] | −6.859 | <.001 |

*Note.* Dependent variable: change in compassion competence (T5 − T1). The model includes all predictors in Table 4 plus T1 compassion competence. R² = 0.2202, adjusted R² = 0.1780, F(17, 314) = 5.22, p < .001. The negative coefficient for T1 compassion competence indicates larger increases among nurses with lower baseline scores (consistent with regression toward the mean). Reference categories as in Table 4. Unlike Table 4, clinical experience in the current unit was entered in years and Δ work loss on the 0–100 scale; the coefficients for these two terms are therefore scaled differently from those in Table 4.

**Table S4.** Importance and stability of SHAP summaries across 500 fold-specific models (100 repetitions of 5-fold cross-validation)

| **Feature** | **XGBoost Share % (dir.)** | **XGBoost Top-5 %** | **XGBoost Dir. agree %** | **XGBoost Signed-sign agree %** | **Random Forest Share % (dir.)** | **Random Forest Top-5 %** | **Random Forest Dir. agree %** | **Random Forest Signed-sign agree %** | **LightGBM Share % (dir.)** | **LightGBM Top-5 %** | **LightGBM Dir. agree %** | **LightGBM Signed-sign agree %** |
| --- | --- | --- | --- | --- | --- | --- | --- | --- | --- | --- | --- | --- |
| Δ NPHA | 21.6 (+) | 99 | 100 | 51 | 19.6 (+) | 98 | 100 | 71 | 18.7 (+) | 94 | 100 | 53 |
| Δ SRA | 16.0 (−) | 95 | 100 | 51 | 14.9 (−) | 92 | 100 | 40 | 14.3 (−) | 95 | 100 | 39 |
| Δ NFQC | 8.3 (+) | 69 | 97 | 57 | 13.3 (+) | 88 | 100 | 40 | 8.9 (+) | 70 | 98 | 62 |
| MS_2 | 12.8 (−) | 57 | 99 | 38 | 8.0 (−) | 32 | 100 | 32 | 9.6 (−) | 31 | 100 | 56 |
| Δ CNPR | 8.4 (+) | 58 | 100 | 46 | 10.5 (+) | 77 | 100 | 62 | 9.6 (+) | 67 | 100 | 59 |
| Δ Work loss | 7.0 (−) | 38 | 79 | 75 | 7.4 (−) | 30 | 82 | 65 | 8.7 (±) | 42 | — | 85 |
| Clinical experience | 8.3 (−) | 41 | 96 | 58 | 4.6 (−) | 18 | 91 | 42 | 8.2 (−) | 41 | 96 | 59 |
| Δ Sleep quality | 5.5 (±) | 25 | — | 42 | 6.4 (+) | 25 | 77 | 62 | 7.4 (±) | 32 | — | 53 |
| Age | 5.3 (−) | 16 | 92 | 62 | 7.5 (−) | 37 | 96 | 45 | 5.6 (−) | 16 | 95 | 58 |
| Δ NMALS | 1.7 (−) | 0 | 79 | 57 | 2.0 (−) | 0 | 66 | 41 | 4.6 (−) | 4 | 73 | 49 |
| MS_3 | 1.4 (+) | 2 | 88 | 54 | 2.7 (+) | 3 | 100 | 50 | 3.3 (+) | 6 | 90 | 42 |
| RA_2 | 1.9 (−) | 1 | 67 | 37 | 1.8 (−) | 0 | 87 | 51 | 0.7 (−) | 0 | 72 | 47 |
| Sex | 1.5 (+) | 0 | 83 | 68 | 0.3 (+) | 0 | 85 | 49 | 0.0 (±) | 0 | — | 100 |
| RA_3 | 0.3 (+) | 0 | 71 | 50 | 0.8 (+) | 0 | 93 | 28 | 0.0 (±) | 0 | — | 100 |
| RA_4 | 0.1 (−) | 0 | 54 | 45 | 0.3 (−) | 0 | 92 | 75 | 0.3 (−) | 0 | 10 | 8 |
| MS_4 | 0.0 (±) | 0 | — | 37 | 0.1 (−) | 0 | 89 | 84 | 0.0 (±) | 0 | — | 100 |

*Note.* Share = mean |SHAP| of the term / sum of mean |SHAP| across 16 terms × 100 (full-sample model). Dir. = sign of the Spearman correlation between feature values and SHAP values (± if |ρ| < 0.10). Top-5 % = percentage of the 500 fold-specific training models in which the term ranked among the five largest mean |SHAP| values. Dir. agree % = percentage of fold-specific models in which the feature–SHAP direction matched the full-sample direction. Signed-sign agree % = percentage of fold-specific models in which the sign of the mean signed SHAP value matched the full-sample sign (shown to illustrate the instability of mean signed SHAP values). Sum of |mean signed SHAP| as a percentage of sum of mean |SHAP|: XGBoost 4.5%, Random Forest 3.4%, LightGBM 3.1%. Δ denotes T5 − T1 change scores; MS/RA reference categories: remained married and maintained religious affiliation.
